# Legionnaires’ Disease in South Africa, 2012–2014

**DOI:** 10.3201/eid2201.150972

**Published:** 2016-01

**Authors:** Nicole Wolter, Maimuna Carrim, Cheryl Cohen, Stefano Tempia, Sibongile Walaza, Philip Sahr, Linda de Gouveia, Florette Treurnicht, Orienka Hellferscee, Adam L. Cohen, Alvaro J. Benitez, Halima Dawood, Ebrahim Variava, Jonas M. Winchell, Anne von Gottberg

**Affiliations:** National Institute for Communicable Diseases, Johannesburg, South Africa (N. Wolter, M. Carrim, C. Cohen, S. Tempia, S. Walaza, P. Sahr, L. de Gouveia, F. Treurnicht, O. Hellferscee, A. von Gottberg);; University of the Witwatersrand, Johannesburg (N. Wolter, M. Carrim, C. Cohen, S. Walaza, L. de Gouveia, A. von Gottberg);; US Centers for Disease Control and Prevention, Pretoria, South Africa (S. Tempia);; University of Pretoria, Pretoria (P. Sahr);; US Centers for Disease Control and Prevention, Atlanta, Georgia, USA (A.L. Cohen, A.J. Benitez, J.M. Winchell);; Pietermaritzburg Metropolitan Hospitals, Pietermaritzburg, South Africa (H. Dawood);; University of KwaZulu-Natal, Pietermaritzburg (H. Dawood);; Klerksdorp-Tshepong Hospital Complex, Klerksdorp, South Africa (E. Variava)

**Keywords:** *Legionella* spp., Legionnaires’ disease, pneumonia, South Africa, Legionellosis, legionella, bacteria, respiratory infections

## Abstract

During June 2012–September 2014, we tested patients with severe respiratory illness for *Legionella* spp. infection and conducted a retrospective epidemiologic investigation. Of 1,805 patients tested, *Legionella* was detected in samples of 21 (1.2%); most were adults who had HIV or tuberculosis infections and were inappropriately treated for *Legionella*.

Data are limited regarding prevalence of *Legionella* spp. bacteria that cause community-acquired pneumonia (CAP) in Africa (*1*), despite the high prevalence of HIV-infected adults in many African countries, including South Africa (*2*). Legionellosis is a notifiable disease in South Africa but is rarely reported. We sought to determine the prevalence of *Legionella* spp. infections in South Africa and describe epidemiologic characteristics of patients with Legionnaires’ disease (LD).

## The Study

During June 2012–September 2014, we conducted a prospective, hospital-based, observational study as part of the severe respiratory illness (SRI) surveillance at 2 sites in South Africa: Klerksdorp-Tshepong Hospital Complex, Klerksdorp, North West Province; and Edendale Hospital, Pietermaritzburg, KwaZulu-Natal Province. A patient with SRI was defined as a person hospitalized with lower respiratory tract infection of any duration. We used a standardized questionnaire to collect demographic and clinical information. Nasopharyngeal specimens and induced sputum samples were tested for *Legionella* spp. infections by using a real-time PCR assay, as previously described (*3*). Specimens that were *Legionella* positive were also tested by real-time PCR assays to identify *L. pneumophila* and *L. longbeachae*. In addition, patients’ specimens were tested for other respiratory pathogens and for HIV. Of the 22 *Legionella*-positive patients, we could trace 17 with whom we conducted a retrospective epidemiologic investigation, which included interviews (detailed study methods in the Technical Appendix).

During June 2012–September 2014, a total of 4,525 SRI patients were enrolled; induced sputum specimens, the recommended specimen type for *Legionella* spp. detection, were collected from 1,805 (40%). Of 1,803 patients with sputum specimens for which data were available, 885 (49%) were male, and 324 (18%) were children <5 years of age. HIV prevalence was 64% (1,025 of 1,594 patients with sputum specimens and known HIV status), and prevalence of active tuberculosis (TB) infection was 24% (421 of 1,758 patients with sputum specimens and known TB status). Of 1,720 patients with sputum specimens and known survival status, 142 (8%) patients died.

**Figure 1:**
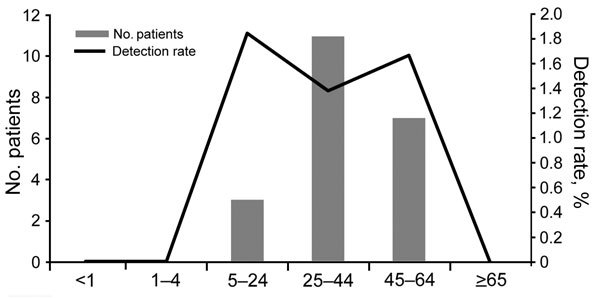
Number of case-patients and detection rate for *Legionella* spp. infections, by age group, South Africa, June 2012–September 2014 (N = 1,803).

Among the 1,805 patients with sputum samples, 21 (1.2%, 95% CI 0.7%–1.7%) tested positive for *Legionella* spp. by real-time PCR. For 1 patient (designated E1 in the online Technical Appendix Table) from whom sputum could not be collected, *Legionella* spp. infection was detected in the nasopharyngeal specimen, so 22 patients with *Legionella* spp. infections were detected in total. Among the 21 patients whose sputum tested positive for *Legionella* spp. infections, median age was 40 years (range 19–59 years; Figure 1), and 11 (52%) were males.

**Figure 2:**
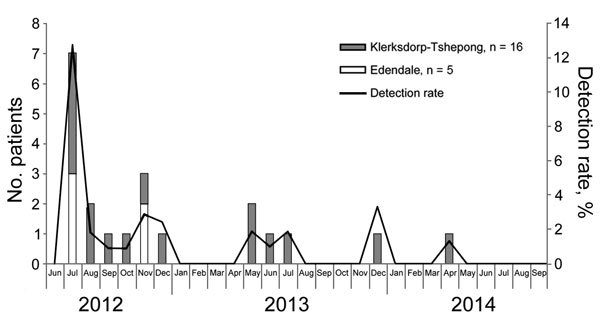
Number of case-patients and detection rate of *Legionella* spp. infections, by month and year, for Edendale Hospital and Klerksdorp-Tshepong Hospital Complex, South Africa, June 2012–September 2014 (N = 1,805). \

A cluster of case-patients (15/21 [71%]) was observed during July–December 2012 (Figure 2), including all 6 from Edendale Hospital and 10 (10/16, 63%) from Klerksdorp-Tshepong Hospital Complex. These sites are geographically distant (≈600 km) from one another, so the respective clusters or outbreaks are unlikely to be related. We did not culture samples with *Legionella* spp. infection, so we were unable to perform strain typing to confirm whether the clusters were caused by related strains. The remaining 6 patients from Klerksdorp-Tshepong Hospital Complex appeared to have sporadic infections.

*Legionella* patients resided in different areas or communities within the cities of Pietermaritzburg and Klerksdorp. Epidemiologic investigation revealed exposure to several potential sources of infection, such as waste management, air conditioners, plumbing, mining, and swimming pools; however, no common exposure could be identified, so environmental sampling and testing were not performed.

Fifteen (75%) of 20 *Legionella* patients with known HIV status were infected with HIV, and 9 (43%) of the 21 patients tested positive for TB at the admission during which *Legionella* infection was detected. HIV or TB infection, or both, was detected in 18 (90%) of 20 patients with known HIV and TB status. A history of active TB before the admission during which *Legionella* was detected was reported for 14 (82%) of 17 patients. For 17 *Legionella* spp.–infected patients for whom information was available, additional LD-associated factors included regular alcohol consumption (10 [59%]), cigarette smoking (9 [53%]), asthma (2 [12%]), and heart disease (2 [12%]).

Eighteen (86%) of 21 patients had symptoms >7 days before hospital admission, a delay possibly occurring because many patients were chronically ill (75% were HIV infected and >43% had TB). Median duration of hospitalization for *Legionella* patients was 4 days (range 1–35 days), and 1 (9%) patient was admitted to intensive care and survived the illness; 4 (20%) patients died. Antimicrobial drug treatment (in-hospital and discharge medication) was known for 21 patients and included amoxicillin/clavulanic acid (16 [76%]), anti-TB medications (15 [71%]), cotrimoxazole (7 [33%]), cefuroxime/ceftriaxone (5 [24%]), and erythromycin (5 [24%]).

*Legionella* spp. isolates were identified for 2 patients as *L. pneumophila* serogroup 1 and *L. longbeachae*. Species could not be determined for 19 patients because of low bacterial loads in their specimens. Of the 21 patients with *Legionella-*positive sputum specimens, co-infections were detected in 14 (67%). Co-infecting pathogens were *Mycobacterium tuberculosis* (9 [43%]), rhinovirus (6 [29%]), respiratory syncytial virus (2 [10%]), adenovirus (2 [10%]), *Bordetella pertussis* (1 [5%]), and *Streptococcus pneumoniae* (1 [5%]).

*Legionella* spp. detection rates in this study were similar to those described in other countries (*4*). However, age distribution tended toward younger adults, not the elderly, the population previously reported as most affected (*4*). Men and women were evenly distributed in our study, although a substantial male predominance is common for LD (*2**,**4*). Differences in age and gender distributions, compared with distributions in other studies, likely result from high HIV and TB prevalence among younger adults in our study population. LD is typically associated with summer because warm and wet conditions promote bacterial replication (*2**,**4*). Longer periods of surveillance are needed to establish seasonality of LD in South Africa.

Clinically, patients with LD in this study were likely to be HIV-infected, chronically ill persons with suspected or confirmed TB and were therefore usually treated for TB infection and discharged. HIV-induced immune suppression and lung damage because of biologic or chemical agents likely increased their susceptibility to *Legionella* infections. Cases of LD and TB occurring simultaneously have been previously described (*5**–**7*). *Legionella* infection in populations with HIV or TB co-infections may cause acute exacerbation of respiratory symptoms, prompting patients to seek hospital care.

In South Africa, treatment for CAP is usually penicillin or ampicillin for adults <65 years of age and amoxicillin/clavunate or cefuroxime for elderly or HIV-infected adults (*8*). However, treatment for LD should include a macrolide or fluoroquinolone (*4*). Only one fourth of *Legionella* patients in this study received appropriate treatment, likely because of clinical inability to distinguish LD from other forms of pneumonia and because clinicians rarely consider *Legionella* when they lack access to diagnostic testing and local prevalence data. This problem is further compounded by the high prevalence of HIV and TB in South Africa. Anti-TB treatment, which was administered to more than two thirds of the *Legionella* patients, would have had therapeutic benefits; rifampin has been shown to have activity against *Legionella* spp. (*9**,**10*). However, suboptimal treatment of *Legionella* patients with co-infections likely contributed to a case-fatality ratio (20%) more than twice that for all SRI patients (8%) (*4**,**11*). Lack of appropriate treatment of patients with CAP in South Africa for atypical pathogens has been described (*12*).

## Conclusions

In South Africa, patients with LD often have chronic illness caused by co-infections such as HIV and TB at time of admission. *Legionella* infections in most patients were undiagnosed, and patients were suboptimally treated for TB or more typical causes of CAP. Increased awareness and improved diagnostic testing could result in earlier diagnosis, appropriate treatment, and improved outcomes for these patients. In addition to routine diagnostics, surveillance for LD should be performed on an ongoing basis for rapid identification and response to outbreaks.

Technical Appendix We conducted a prospective, hospital-based, observational study as part of Severe Respiratory Illness (SRI) surveillance from June 2012–September 2014 at 2 sites in South Africa: Klerksdorp-Tshepong Hospital Complex, Klerksdorp, North West Province; and Edendale Hospital, Pietermaritzburg, KwaZulu-Natal Province.
