# Supplementary material for: Legionnaires’ Disease in South Africa, 2012–2014
Source: Emerg Infect Dis. 2016 Jan;22(1):131–3. doi: 10.3201/eid2201.150972 (PMC4696697; doi:10.3201/eid2201.150972)
Supplement: Technical Appendix — We conducted a prospective, hospital-based, observational study as part of Severe Respiratory Illness (SRI) surveillance from June 2012–September 2014 at 2 sites in South Africa: Klerksdorp-Tshepong Hospital Complex, Klerksdorp, North West Province; and Edendale Hospital, Pietermaritzburg, KwaZulu-Natal Province. [file 15-0972-Techapp-s1.pdf]

Article DOI: http://dx.doi.org/10.3201/eid2201.150972

Legionnaires' Disease in South Africa, 2012–2014

Technical Appendix

Materials and Methods

Study Design

We conducted a prospective, hospital-based, observational study as part of Severe Respiratory Illness (SRI) surveillance from June 2012–September 2014 at 2 sites in South Africa: Klerksdorp-Tshepong Hospital Complex, Klerksdorp, North West Province; and Edendale Hospital, Pietermaritzburg, KwaZulu-Natal Province.

A case of SRI was defined as a person hospitalized with symptoms of any duration and meeting age-specific clinical inclusion criteria as follows: children from 2 days to <3 months of age with physician-diagnosed sepsis or lower respiratory tract infection (LRTI); children from 3 months to <5 years of age with physician-diagnosed LRTI; and patients ≥5 years of age who meet a modified World Health Organization case definition for SRI (*1*): 1) fever (>38°C) or reported fever; 2) cough or sore throat; and 3) shortness of breath or difficulty breathing, with or without clinical or radiographic findings of pneumonia. In addition, we enrolled patients with a clinical diagnosis of suspected tuberculosis (TB). Chest x-rays are not performed routinely on LRTI patients at the site hospitals, so radiologic confirmation of pneumonia could not be obtained. A case of Legionnaires' disease was defined as a person hospitalized with SRI and positive results for *Legionella* spp. infection determined by PCR from an induced sputum or nasopharyngeal (NP) specimen. A standardized questionnaire was used to collect demographic and clinical information, such as age, sex, regular alcohol or cigarette use, mining exposure, underlying illness, prior antimicrobial drug use, duration of symptoms, length of hospitalization, in-hospital antimicrobial drug treatment, treatment with supplemental oxygen, admission to an intensive care unit, and outcome.

Specimen Collection

NP, induced sputum, and whole blood samples were collected. NP specimens, including combined NP and oropharyngeal swabs (FLOQSwabs, Copan Diagnostics, Murrieta, CA, USA) for patients ≥5 years of age or NP aspirates for children <5 years of age were placed in universal transport medium (Copan Diagnostics) and transported at 4°C to the National Institute for Communicable Diseases, Johannesburg, South Africa. As of July 2013, sputum samples were stored at −20°C and transported on dry ice.

Detection of *Legionella* spp.

Sputum samples were digested with dithiothreitol (Roche Diagnostics, Mannheim, Germany). Total nucleic acids were extracted from 200 µL of sample by using the MagNA Pure 96 instrument (Roche) and MagNA Pure 96 DNA and Viral NA SV kit (Roche). Nucleic acids were eluted into 100 µL of elution buffer and stored at −20°C.

NP and induced sputum samples were tested for *Legionella* spp. by using a multiplex real-time PCR assay that also detected *Mycoplasma pneumoniae, Chlamydia pneumoniae*, and the human *RNaseP* gene (*2*). Twenty-five µL reactions were performed by using PerfeCTa Multiplex qPCR Supermix (Quanta Biosciences, Gaithersburg, MD, USA), 6.5 µL DNA, and primers and probes, as previously described (*2*). Samples with a C_t-value >45 were recorded as negative. PCR was performed by using an Applied Biosystems 7500 Fast real-time PCR instrument (Thermo-Fisher Scientific, Waltham, MA).

Legionella spp. Identification

Legionella-positive specimens were further tested in 2 real-time PCR assays. The first assay confirmed *Legionella* spp. and identified *L. pneumophila* and *L. pneumophila* serogroup 1 (*3*). The second assay identified *L. longbeachae*. The reaction mix comprised of 12.5 µL of PerfeCTa Multiplex qPCR Supermix (Quanta Biosciences), 5 µL DNA, 25 µmol/L primers (LLB-F –TGGTTTTCGAAATCATCAGTATGC, LLB-R-CTGTCTAAAACACTTCTCTCCCGATA) and 5 µM probe (Quasar670-TTTAATTTAGTTCCCACCAGCAAGGATGGC-BHQ3) made to a final volume of 25 µL. Cycling conditions were as follows: 1 cycle of 95°C for 10 min, followed by 50 cycles of 95°C for 15 s and 60°C for 1 min.

TB Testing

TB testing was conducted at the site laboratory; additional testing was done at the NICD. Sputum samples were tested for *Mycobacterium tuberculosis* by smear microscopy,

culture or PCR. Tests were performed as follows: smear microscopy by using fluorescence auramine staining for acid-fast bacilli; liquid media by using BD Bactec MGIT 960 (Beckton, Dickinson, Franklin Lakes, NJ, USA) for culture; and TB PCR by using the Xpert MTB/RIF system (Cepheid, Sunnyvale, CA, USA). Positive cultures were identified as *M. tuberculosis* complex by using Ziehl-Neelsen staining and antigen testing.

Detection of Other Respiratory Pathogens

SRI patients were tested for additional respiratory pathogens. NP specimens were tested for specific respiratory viruses (i.e., influenza, adenovirus, enterovirus, rhinovirus, human metapneumovirus, respiratory syncytial virus, and parainfluenza types 1–3) by multiplex real-time PCR (*4*). NP and induced sputum specimens were tested for *Bordetella pertussis* (*5*), and blood specimens were tested for *Streptococcus pneumoniae* (*6*) and *Haemophilus influenzae* (*7*).

Determination of HIV Status

HIV results were obtained from a combination of 2 sources: patient clinical records when available and for consenting patients, a blind, linked dried-blood spot tested at NICD. For patients for whom both results were available, the NICD result was used. Testing included HIV ELISA testing for patients ≥18 months of age and PCR testing for children <18 months of age.

Epidemiologic Case Investigation

An epidemiologic investigation was conducted retrospectively for *Legionella*-positive patients who could be traced and included collection of additional information from the patient or a close relative for a deceased patient. Additional information included potential *Legionella* exposure, past medical history, factors associated with disease, and discharge medication.

Statistical Analysis

As sputum specimens are the optimal specimen type for *Legionella* spp. detection, analyses were performed for patients who had a sputum sample collected. Detection rate was defined as the number of *Legionella* case-patients among all SRI case-patients with sputum samples collected and tested.

Ethical Approval

The protocol was approved by the Universities of the Witwatersrand (M081042) and KwaZulu-Natal (BF157/08). The U.S. Centers for Disease Control and Prevention deemed the study a non-research, surveillance activity.

References

1. World Health Organization. WHO interim global epidemiological surveillance standards for influenza. Geneva: The Organization; 2012 [cited 2013 Feb 14]. http://www.who.int/influenza/resources/documents/INFSURVMANUAL.pdf

2. Thurman KA, Warner AK, Cowart KC, Benitez AJ, Winchell JM. Detection of *Mycoplasma pneumoniae*, *Chlamydia pneumoniae*, and *Legionella spp*. in clinical specimens using a single-tube multiplex real-time PCR assay. Diagn Microbiol Infect Dis. 2011;70:1–9. http://dx.doi.org/10.1016/j.diagmicrobio.2010.11.014

3. Benitez AJ, Winchell JM. Clinical application of a multiplex real-time PCR assay for simultaneous detection of *Legionella* species, *Legionella pneumophila*, and *Legionella pneumophila* serogroup 1. J Clin Microbiol. 2013;51:348–51. http://dx.doi.org/10.1128/JCM.02510-12

4. Pretorius MA, Madhi SA, Cohen C, Naidoo D, Groome M, Moyes J, et al. Respiratory viral coinfections identified by a 10-plex real-time reverse-transcription polymerase chain reaction assay in patients hospitalized with severe acute respiratory illness–South Africa, 2009–2010. J Infect Dis. 2012;206(Suppl 1):S159–65. http://dx.doi.org/10.1093/infdis/jis538

5. Tatti KM, Sparks KN, Boney KO, Tondella ML. Novel multitarget real-time PCR assay for rapid detection of *Bordetella* species in clinical specimens. J Clin Microbiol. 2011;49:4059–66. http://dx.doi.org/10.1128/JCM.00601-11

6. Carvalho M, Tondella ML, McCaustland K, Weidlich L, McGee L, Mayer LW, et al. Evaluation and improvement of real-time PCR assays targeting *lytA*, *ply*, and *psaA* genes for detection of pneumococcal DNA. J Clin Microbiol. 2007;45:2460–6. http://dx.doi.org/10.1128/JCM.02498-06

7. Maaroufi Y, De Bruyne JM, Heymans C, Crokaert F. Real-time PCR for determining capsular serotypes of *Haemophilus influenzae*. J Clin Microbiol. 2007;45:2305–8. http://dx.doi.org/10.1128/JCM.00102-07

Technical Appendix Table. Demographic, epidemiologic, and clinical characteristics of patients positive for *Legionella*, South Africa, June 2012–September 2014 (N = 22)*

Case no.	Hospital, admission date	Sex, age, y	Symptom duration, d†	Potential exposure to contaminated water source	HIV	TB‡	Risk factors§	Hospital duration, d (treatment¶)	Outcome
E1#	Edendale, 28 Jun 2012	M, 56	4	Worked/lived at waste management compound, compost exposure	Unk	Sput unav	Heart disease, alcohol	35 (TB, amoxicillin-clavunate)	Survived
E2**	Edendale, 3 Jul 2012	M, 52	Unk	Unk	Unk	Sput –; treated for TB in past year	None	6 (Amoxicillin-clavunate)	Died
E3**	Edendale, 9 Jul 2012	F, 55	19	Unk	+	Sput +	None	3 (Amoxicillin-clavunate)	Died
E4	Edendale,16 Jul 2012	M, 20	13	Attended school	–	Sput –	Former smoker, alcohol	22 (TB, amoxicillin-clavunate)	Survived
E5**	Edendale, 6 Nov 2012	F, 41	66	Unk	+	Sput –	None	6 (Cefuroxime)	Survived
E6	Edendale, 13 Nov 2012	F, 32	8	Cleaner at hospital/air conditioner maintenance	+	Sput +	None	7 (TB, amoxicillin-clavunate, cefuroxime)	Survived
T1**	Tshepong, 3 Jul 2012	F, 34	30	Unk	+	Sput +	None	1 (HIV,TB, amoxicillin-clavunate, cotrimoxazole)	Died out of hospital
T2**	Tshepong, 4 Jul 2012	F, 46	30	Unk	+	Sput –	None	5 (TB)	Survived
T3	Tshepong, 10 Jul 2012	F, 32	61	None known	+	Sput +	Heart disease	2 (HIV, TB, ceftriaxone, cefuroxime)	Survived
T4	Tshepong, 30 Jul 2012	M, 44	1	Gardening/ plumbing work, compost exposure	–	Sput –	Current smoker, alcohol	2 (TB, erythromycin)	Survived
T5	Tshepong, 14 Aug 2012	M, 34	10	Mining, gardening, renovation work	+	Sput +	Asthma, former smoker, alcohol	1 (HIV, erythromycin, amoxicillin-clavunate)	Survived
T6	Tshepong, 16 Aug 2012	F, 19	13	None known	–	Sput +	Alcohol	3 (TB, amoxicillin-clavunate)	Survived
T7	Tshepong, 20 Sep 2012	F, 50	Unk	None known	+	Sput –	Asthma, current smoker (snuff), alcohol	5 (HIV, TB, cotrimoxazole, erythromycin, amoxicillin-clavunate)	Survived
T8	Tshepong, 21 Oct 2012	M, 25	14	Sewage exposure	–	Sput +	Alcohol	1 (TB, amoxicillin-clavunate, cefuroxime)	Survived
T9	Tshepong, 5 Nov 2012	F, 39	188	None known	+	Sput –	Current smoker (snuff)	11 (HIV, TB, cotrimoxazole)	Survived
T10	Tshepong, 6 Dec 2012	M, 55	21	None known	+	Sput –	Former smoker	1 (TB, amoxicillin-clavunate, cefuroxime, cotrimoxazole, erythromycin)	Survived
T11	Tshepong, 6 May 2013	F, 52	22	Hospital admission 22 Apr 2013–1 May 2013	+	Sput –	None	3 (HIV, cotrimoxazole, amoxicillin-clavunate)	Died
T12	Tshepong, 9 May 2013	M, 59	13	Visited wife in hospital Apr 2013	+	Sput –	Current smoker, alcohol	5 (Amoxicillin-clavunate)	Survived
T13	Tshepong, 11 Jun 2013	M, 42	10	Building and cleaning of dams/ swimming pools	+	Sput +	Former smoker, alcohol	Unk (HIV, TB, amoxicillin-clavunate,	Survived

Case no.	Hospital, admission date	Sex, age, y	Symptom duration, d†	Potential exposure to contaminated water source	HIV	TB‡	Risk factors§	Hospital duration, d (treatment¶)	Outcome
								cotrimoxazole)	
T14	Tshepong, 1 Jul 2013	M, 22	2	Home renovation Jan–Mar 2013	–	Sput +	Former smoker	2 (TB, amoxicillin-clavunate)	Survived
T15	Tshepong, 5 Dec 2013	M,38	3	None known	+	Sput –	Current smoker, alcohol	4 (TB, cotrimoxazole, amoxicillin-clavunate)	Survived
T16	Tshepong, 16 Apr 2014	M, 38	13	Mining exposure	+	Sput –	Former smoker	27 (HIV, erythromycin)	Survived

*None known, patient or family member did not report exposure to any water source listed on questionnaire; TB, *Mycobacterium tuberculosis* infection; Unk, unknown: information could not be obtained because interviews could not be conducted; +, positive; –, negative; unav, unavailable; Sput, sputum.
†Symptom duration includes time from symptom onset until hospital admission
‡TB status was determined by sputum test with positive (Sput +) or negative (Sput –) results. Sputum test was unavailable for patient E1.
§Risk factors assessed were asthma, lung disease, kidney disease, heart disease, liver disease, diabetes, cancer, emphysema, former/current cigarette smoking, and current alcohol consumption.
¶Includes in-hospital and discharge treatment, mostly for HIV or TB infection or both.
#*Legionella* spp. identified with nasopharyngeal specimen.
**Retrospective epidemiologic investigation not possible because patient died or patient or family member could not be traced.
